# The contribution of spatial mass effects to plant diversity in arable fields

**DOI:** 10.1111/1365-2664.13414

**Published:** 2019-06-03

**Authors:** Helen Metcalfe, Kirsty L. Hassall, Sébastien Boinot, Jonathan Storkey

**Affiliations:** ^1^ Sustainable Agricultural Sciences Rothamsted Research Harpenden Hertfordshire UK; ^2^ Computational and Analytical Sciences Rothamsted Research Harpenden UK; ^3^ System, INRA, CIHEAM‐IAMM, Montpellier SupAgro Univ Montpellier Montpellier France

**Keywords:** agricultural landscape, arable fields, conservation headlands, fidelity score, field edge, plant diversity, spatial mass effects, weeds

## Abstract

1. In arable fields, plant species richness consistently increases at field edges. This potentially makes the field edge an important habitat for the conservation of the ruderal arable flora (or ‘weeds’) and the invertebrates and birds it supports. Increased diversity and abundance of weeds in crop edges could be owing to either a reduction in agricultural inputs towards the field edge and/or spatial mass effects associated with dispersal from the surrounding landscape.
2. We contend that the diversity of weed species in an arable field is a combination of *resident* species, that can persist under the intense selection pressure of regular cultivation and agrochemical inputs (typically more ruderal species), and *transient* species that rely on regular dispersal from neighbouring habitats (characterised by a more ‘competitive’ ecological strategy).
3. We analysed a large dataset of conventionally managed arable fields in the UK to study the effect of the immediate landscape on in‐field plant diversity and abundance and to quantify the contribution of spatial mass effects to plant diversity in arable fields in the context of the ecological strategy of the resulting community.
4. We demonstrated that the decline in diversity with distance into an arable field is highly dependent on the immediate landscape, indicating the important role of spatial mass effects in explaining the increased species richness at field edges in conventionally managed fields.
5. We observed an increase in the proportion of typical arable weeds away from the field edge towards the centre. This increase was dependent on the immediate landscape and was associated with a higher proportion of more competitive species, with a lower fidelity to arable habitats, at the field edge.
6. *Synthesis and applications*. Conserving the ruderal arable plant community, and the invertebrates and birds that use it as a resource, in conventionally managed arable fields typically relies on the targeted reduction of fertilisers and herbicides in so‐called ‘conservation headlands’. The success of these options will depend on the neighbouring habitat and boundary. They should be placed along margins where the potential for ingress of competitive species, that may become dominant in the absence of herbicides, is limited. This will enhance ecosystem services delivered by the ruderal flora and reduce the risk of competitive species occurring in the crop.

## Abstract 2

Conserving the ruderal arable plant community, and the invertebrates and birds that use it as a resource, in conventionally managed arable fields typically relies on the targeted reduction of fertilisers and herbicides in so‐called ‘conservation headlands’. The success of these options will depend on the neighbouring habitat and boundary. They should be placed along margins where the potential for ingress of competitive species, that may become dominant in the absence of herbicides, is limited. This will enhance ecosystem services delivered by the ruderal flora and reduce the risk of competitive species occurring in the crop.

## 1 INTRODUCTION

Arable field edges (defined as the first few metres of crop by Marshall & Moonen, 2002) have often been observed to support higher levels of species richness than field centres (Alignier, Petit, & Bohan, 2017; Marshall, 1989; Wilson & Aebischer, 1995). This increased weed diversity at field edges presents a potential opportunity to support the conservation of biodiversity on farmland (Albrecht, Cambecèdes, Lang, & Wagner, 2016, Fried, Petit, Dessaint, & Reboud, 2009) and reconcile the trade‐off between biodiversity and agricultural productivity (the increase in plant diversity observed on organic farms is largely made up of species found in the cropped areas; Fuller et al., 2005). A diverse, abundant, naturally regenerated arable flora has been demonstrated to make a disproportionate contribution to supporting other trophic levels, including phytophagous insects (Storkey et al., 2013), bees (Bretagnolle & Gaba, 2015), natural enemies (Brooks et al., 2012; Norris & Kogan, 2000) and birds (Eraud et al., 2015; Henderson et al., 2012). However, the potential of the field edge flora to provide these resources will depend on the relative importance of two different processes that explain the increased diversity at crop edges.

First, it is often argued that field edges represent a valuable habitat for arable plants exhibiting typical ruderal traits (sensu Grime, 1974) due to a decrease in the intensity of fertiliser and herbicide inputs at the field edge (Alignier et al., 2017; Marshall & Moonen, 2002; Wilson & Aebischer, 1995). However, this is often inferred and has rarely been directly measured (Tsiouris & Marshall, 1998; Weaver, Downs, & Thomas, 2005). This distinct habitat is said to support ruderal species that have an affinity to arable fields due to an adaptation to fertile and disturbed environments (Bourgeois et al., 2018) yet can no longer sustain populations under intensive herbicide and fertiliser pressure in the centre of the field (Fried et al., 2009; Kleijn & Van der Voort, 1997; Wagner, Bullock, Hulmes, Hulmes, & Pywell, 2017). In this scenario, the field edge also provides an opportunity for threatened arable plants to persist (Fried et al., 2009) as well as supporting the delivery of ecosystem services through enhanced biodiversity in agricultural landscapes (Bretagnolle & Gaba, 2015; Marshall et al., 2003; Storkey & Westbury, 2007). Conservation headlands (areas of crop at the edge of the field with reduced fertiliser and herbicide inputs) are the primary policy mechanism specifically targeted at conserving the arable flora (Albrecht et al., 2016).

Another hypothesis for the increased diversity at field edges is that they are subject to spatial mass effects or spill‐over of species from neighbouring habitats (Shmida & Wilson, 1985). Spatial mass effects augment diversity within mosaic landscapes due to immigration from adjacent habitats (Kunin, 1998). Under this hypothesis, field edges receive the same management as the field centre but are biotically linked to the neighbouring habitat and, therefore, represent a unique habitat (Gabriel, Roschewitz, Tscharntke, & Thies, 2006; Kovar, 1992; Le Coeur, Baudry, Burel, & Thenail, 2002; Marshall & Moonen, 2002). As the agricultural landscape presents very steep transitional gradients between the intensively managed cropped area and the semi‐natural field boundary vegetation, we might expect that such spatial mass effects make a significant contribution to plant diversity and abundance at the edge of fields, due to the close proximity of the contrasting habitats. Under this hypothesis, the increase in plant diversity, through the addition of non‐arable plants, could be considered as a threat to both crop production and the conservation of rare arable plants in field edges, particularly if the additional species have a more competitive ecological strategy.

Here, we aim to determine the contribution of spatial mass effects to plant diversity in arable fields, and the implications for both crop production and the conservation of farmland biodiversity in conventionally managed fields. Previous studies reporting the effect of the landscape on plant species richness, have focussed on large‐scale landscape factors and have shown that landscape heterogeneity or field size can affect weed species richness (Alignier et al., 2017; Gaba, Chauvel, Dessaint, Bretagnolle, & Petit, 2010; Gabriel et al., 2006; Gabriel, Thies, & Tscharntke, 2005; Poggio, Chaneton, & Ghersa, 2010; Roschewitz, Gabriel, Tscharntke, & Thies, 2005). Heterogeneous landscapes are composed of diverse habitat mosaics and so the species pool in these landscapes should be greater as the niche space within these different habitat types is broader than would be found in a homogeneous landscape. Smaller fields, with a higher edge/area ratio, have a greater probability of being colonised from these surrounding habitats. In contrast, other studies have failed to detect a significant relationship between landscape heterogeneity and weed species richness (Alignier et al., 2017; Bohan & Haughton, 2012; Marshall, West, Kleijn, 2006).

One reason for the resulting uncertainty around the contribution of spatial mass effects to plant diversity in arable fields is that spill‐over from neighbouring habitats will depend on the features of the immediate landscape, which are not captured in typically used metrics of habitat heterogeneity (e.g. the proportion of arable land in a predefined radius). Considering that plant mean dispersal distance is 50 m (Nathan, 2006) and that many plants typical of arable landscapes are gravity‐dispersed (Benvenuti, 2007), it is likely that only the habitats immediately adjacent to the crop impact local species richness and the wider landscape exerts a relatively minor influence.

**Figure 1:**
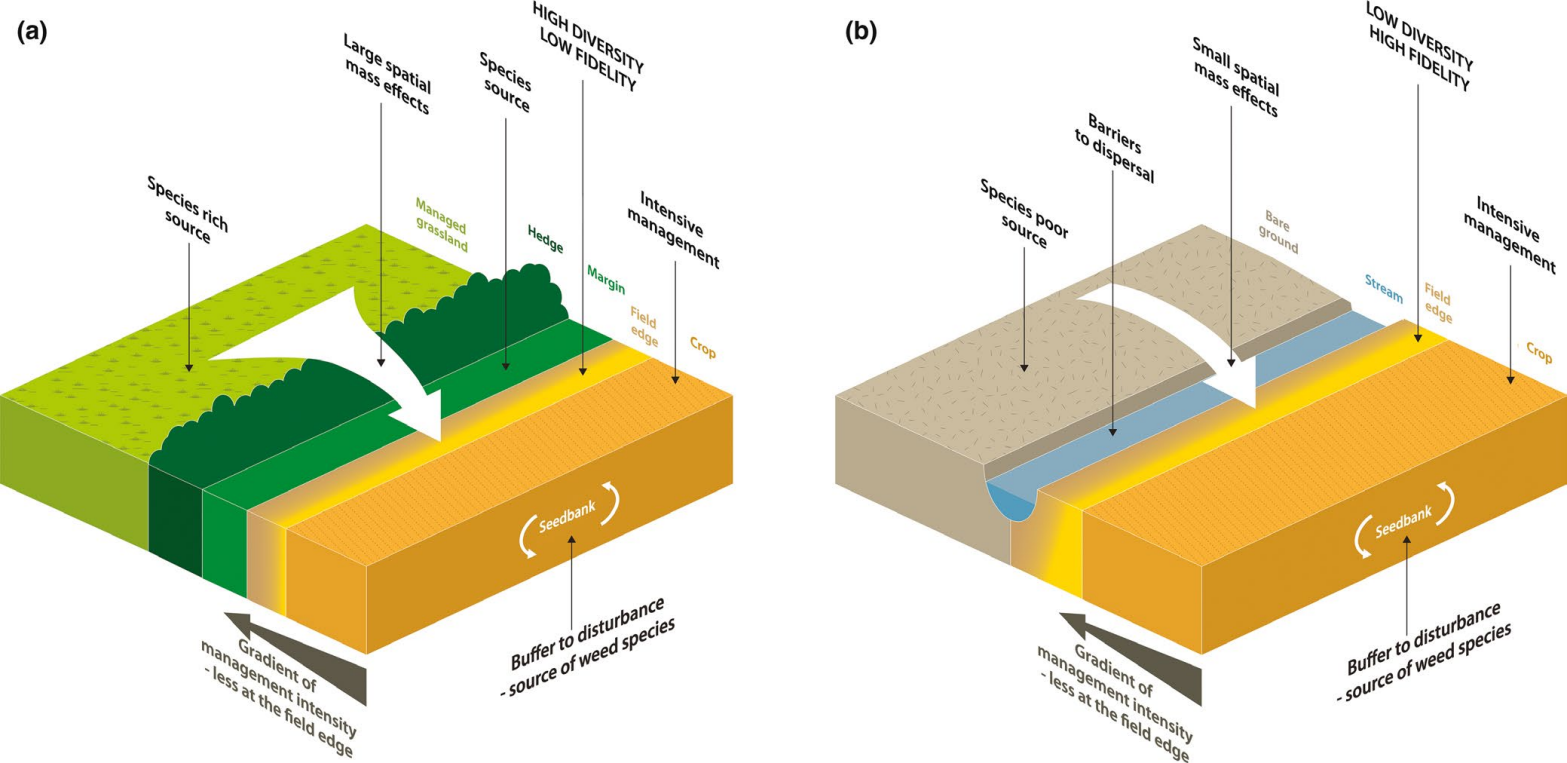
The gradient of management intensity (reduction in fertiliser/herbicide inputs) towards the field edge may account for some increase in species richness observed at field edges. However, spatial mass effects will also contribute to the change in species richness and abundance at field edges. We hypothesise that the size of these effects is relative to the immediately adjacent habitats and field boundaries. (a) Large spatial mass effects, owing to species rich adjacent environments (e.g. managed grassland), boundaries (e.g. hedge) and margin, lead to a greater species richness and more abundance with a larger proportion of transient species in the community. In this scenario, fidelity scores, measuring the affinity of species to the arable habitat, will be lower. (b) Small spatial mass effects, owing to species poor adjacent environments (e.g. bare ground) and boundaries (e.g. water) lead to a low abundance of weeds with a reduced transient community in the field edge. Here, the resident weed community, buffered by the seedbank, will comprise the greater part and so the field edge community will be less diverse and show higher fidelity scores

We present a novel analysis of the dataset from the Farm‐Scale Evaluations (FSE) of genetically modified, herbicide‐tolerant (GMHT) crops that represent the most extensive survey of biological communities in arable fields in the UK (Firbank et al., 2003). The data on weed communities have previously been used to analyse the importance of crop management, crop rotation and landscape in driving variation in field‐scale plant diversity and abundance (Bohan & Haughton, 2012; Bohan et al., 2011; Heard et al., 2003). Here, we develop models of weed diversity and abundance that use previously unreported data on the field boundary, margin (an established strip between the boundary and field edge), and habitat immediately neighbouring each field in the FSE. We contend that field‐scale arable plant diversity is a combination of *resident* species that can persist under the intense selection pressure of cultivation and agrochemical inputs (typical arable species), and *transient* species that rely on regular re‐colonisation from neighbouring habitats, characterised by a more ‘competitive’ ecological strategy (Figure 1). We use an objective measure of fidelity to arable habitats to determine the extent to which plant species are adapted to the arable habitat, and quantify the relative contribution of resident and transient species to a community. We also relate this measure of fidelity to independent measures of competitiveness to indicate the extent to which spatial mass effects at field edges could pose a threat to both crop production and the conservation of ruderal habitats.

If spatial mass effects are making an important contribution to the variance in field‐scale plant diversity, we would expect: (a) a significant interaction between the decline in diversity and abundance with distance into the field and the nature of the neighbouring habitat and/or boundary feature, (b) an increase in fidelity to arable field habitats from the field edge to the centre, and (c) a weakening of these patterns post‐herbicide treatment, as transient species are removed, leaving a greater proportion of resident species that are able to persist in intensively managed fields owing to buffering from large persistent seedbanks or evolved resistance to herbicides. If spatial mass effects are important in determining field edge diversity and transient species are competitive with low fidelity to the arable environment, these species will pose a threat to crop production and the conservation of farmland biodiversity.

## 2 MATERIALS AND METHODS

### 2.1 FSE dataset

We used data collected as part of the FSE study of GMHT crops (Firbank et al., 2003). The study covered 296 fields growing either sugar beet, maize, winter or spring oilseed rape (OSR) and ran from 2000 to 2002. Each field was split into two halves, a GMHT half and a conventionally cropped half. A wide range of metrics of agricultural biodiversity were collected (Firbank et al., 2003). The fields were spread widely across the lowlands of eastern and southern Britain (see Figure S1a) and were broadly representative of contemporary agriculture (Champion et al., 2003). Herbicide treatment was not stipulated in the experimental design for the conventional crops and it was left to farmers to implement ‘cost‐effective’ weed control using their normal farm management practice (Heard et al., 2003). We only used data from the conventionally managed treatments and removed all data from GMHT treatments from our analyses.

### 2.2 Weed data

Weed counts at the species level were done in 0.25 × 0.5 m quadrats on 12 transects in each half‐field. The transects ran perpendicularly from the field edge into the crop with sampling points at 2, 4, 8, 16 and 32 m (Heard et al., 2003; see Figure S1b). Weeds were counted at two separate time points during the year of the experiment. The first count (pre‐herbicide dataset) was made after crop sowing and, where possible, prior to any post‐emergence herbicide application. The second count (post‐herbicide dataset) was done after the last herbicide application, allowing a delay period for mortality to occur. To address the limitation that the original experimental design focused on spring crops, we also considered a third count (winter wheat dataset) which was made in May–June of the year following the experiments for any fields where the growers chose to grow a winter wheat crop (Figure S2).

### 2.3 In‐field and landscape factors

Information about the soil type of each field was provided by the farmers at the site selection stage and grouped into four categories: light, medium, heavy and organic (Firbank et al., 2003). Farmers also provided data on the field size.

The land adjacent to the trial field at the end of each transect was classified into broad habitat categories (Firbank et al., 2003—categories from: Carey et al., 2002; UK Biodiversity Steering Group, 1998). We grouped these landscape factors into a single categorical variable ‘adjacent environment’ with categories including bare ground (ploughed field or urban), crop, managed grassland, natural grassland and woodland (Figure S3). Among the 3,028 transects, 885 were found to have multiple adjacent environments present and so were not included in the subsequent analysis.

Other landscape information was recorded for a 10‐m section at the field edge for each transect position (Firbank et al., 2003). These data included the presence or absence of margins (Figure S4), the width of any margin present and the types of field boundary features (Figure 1). We grouped the boundary attributes into a single categorical variable ‘adjacent boundary’ that could take any one of the following values: ditch, hedge or tree line, road, water course or no boundary (Figure S5). Transects found to have multiple adjacent boundaries, for example both a hedge and a ditch, were not included in the subsequent analysis to avoid rank deficiencies in the modelling step. At this stage, a further 377 transects were removed from the dataset leaving 1,766 transects in our analyses.

### 2.4 Analysis

#### 2.4.1 Diversity indices

For each of the three weed count datasets, we calculated the weed species richness and total weed abundance in each quadrat.

#### 2.4.2 Fidelity scores

Fidelity scores (*F*) are based on the relative observed species frequencies within the habitat of interest (arable fields) compared to other habitats (Equation 1, Chytrý, Tichý, Holt, & Botta‐Dukát, 2002). Fidelity scores range from −1 to +1 with positive (negative) values indicating that the species and the habitat of interest co‐occur more (less) frequently than would be expected by chance. Larger positive values indicate a greater degree of joint fidelity. We defined the habitat of interest to be the central cropped area of arable fields and considered each field site in the FSE to be one plot in the habitat of interest (for the purposes of calculating this metric, we omitted the data in quadrats 2 m from the crop edge to avoid edge effects and to exclude the transient community, *N_p_ = *268). We used independent data from the Countryside Survey (Carey et al., 2008) to represent plots in other habitats. The Countryside Survey covered a total of 591 1 km × 1 km sample squares spread across England, Scotland and Wales, representative of the variations in the climate and geology of the three countries. The species of plant present were recorded for each plot. From these data, we excluded all plots in an arable or horticultural habitat as well as plots from non‐terrestrial habitats, leaving a total of *N =* 16,024 plots.

(1) $$F=\frac{N\cdot n_{p}-n\cdot N_{p}}{\sqrt{n\cdot N_{p}\cdot \left(N-n\right)\cdot \left(N-N_{p}\right)}}.$$

We computed a fidelity score for each of the 181 species present in the FSE dataset using Equation 1 where *n* is the total number of plots in which the species is found (across both the FSE and Countryside Survey datasets) and *n*_p_ is the number of plots where the species is found in the habitat of interest (FSE dataset only).

For each quadrat within the FSE dataset, a community weighted mean (CWM) of fidelity scores (*F*_CWM_) was calculated based on abundance data following

$$F_{\text{CWM}}=\sum_{i=1}^{n}p_{i}F_{i}$$

where *i* is the number of species present in the quadrat, *p_i_* the proportion of the total number of individuals in the quadrat made up by species *i* and *F_i_*the fidelity score for species *i*.

#### 2.4.3 Species competitiveness

We took two complementary approaches to assess the relationship between fidelity and the relative competitiveness of weed species. First, we used data on the competitive index of various weed species estimated by Marshall et al. (2003) as the weed density required to give 5% crop yield loss in wheat, with a *lower value* indicating increased competitiveness. While the absolute value of the index will be specific to a given crop, and will also depend on local environment, weather and management, it is nevertheless a useful quantitative measure of relative competitiveness. For the 25 species for which these data were available, that were also found in the FSE, we compared this competitive index (log values +0.1) to the fidelity score for that species to determine whether these two metrics were correlated (Pearson correlation).

In the second approach we recorded the ecological strategy (C/S/R, and all combinations thereof, where C species are competitors, S species are stress‐tolerators and R species are ruderals) according to Grime, Hodgson, and Hunt (2014), where available, for all species present in the FSE. These strategies are indicative of adaptation to environments with contrasting soil fertility and disturbance with the difference between ruderals and competitors representing a contrast in competitive ability in fertile environments. We determined the average fidelity score for each group and tested for significant differences in fidelity score between the ecological strategies (one‐way ANOVA).

#### 2.4.4 Generalised linear mixed effects models

We investigated the effect of landscape and in‐field factors on weed diversity, abundance, and CWM fidelity scores using generalised linear mixed effects models (GLMMs). Observations at the quadrat scale were used as the response, with sites and transects nested within sites included as random effects following the original experimental design. There was no evidence for spatial structure in any response variable and so no further correlation structure was incorporated into these random effects. Species richness and abundance were assumed to follow a Poisson distribution, while CWM fidelity scores were assumed to follow a normal distribution. For all responses, the canonical link function (natural logarithm for Poisson responses and identity for normal responses) was used. For each Poisson response, the dispersion parameter was estimated to account for over and under dispersion. We considered the following terms in the fixed effects model: distance from field edge (natural logarithms), adjacent environment, adjacent boundary, the presence of a margin and its width, soil type, field size, and crop type. We also included the interaction between each landscape and in‐field factor with distance from the field edge. Due to high levels of imbalance between higher order factor level combinations, in particular the presence of combinations with zero counts (11 out of 157, see Table S1), higher order terms were not considered in the model. Models were fitted using the method of Schall (1991) with terms fitted in the following order: distance + adjacent environment + adjacent boundary + margin/width + soil type + field size + crop type + distance:adjacent environment + distance:adjacent boundary + distance:margin/width + distance:soil type + distance:field size + distance:crop type. Terms were selected using backwards elimination according to the largest *p*‐value given by an approximate *F*‐test when that term was dropped (Kenward & Roger, 1997). The final predictive model was chosen when all remaining terms gave significant values (*p* ≤ 0.05) for an *F* test when dropped from the model.

## 3 RESULTS

### 3.1 Weed diversity and abundance

All three FSE datasets (pre‐herbicide, post‐herbicide and winter wheat) showed similar species richness with a mean of two to three species across all quadrats. Weed abundance was generally lower in the post‐herbicide dataset than in either the pre‐herbicide or winter wheat counts (Table 1).

**Table 1:** Summary statistics of diversity and abundance at the quadrat level across all three weed datasets

Diversity metric	Dataset	Number of quadrats	Mean	Median	Min	Max	Lower quartile	Upper quartile	Variance	Skew
Species richness	Pre‐herbicide	7,407	2.773	2	1	16	1	4	3.387	1.419
	Post‐herbicide	6,886	2.467	2	0	17	1	3	2.346	1.436
	Winter wheat	3,443	2.896	2	1	15	1	4	4.458	1.639
Abundance	Pre‐herbicide	7,407	14.33	6	1	491	2	16	639.4	6.376
	Post‐herbicide	6,886	8.056	5	0	214	2	10	115.9	4.651
	Winter wheat	3,443	15.64	7	1	459	3	18	643.6	5.4

### 3.2 Fidelity

Of the 181 species present in the FSE dataset, the species scoring highest for fidelity were those that are typically considered as weed species with R species achieving the highest fidelity scores (Table 2). The species scoring the lowest for fidelity were mostly perennials, more commonly associated with hedgerows and grass margins, including seedlings of woody species, and were characterised by a more competitive ecological strategy. The absence of species scoring less than −0.1 indicates that there were no species atypical of the arable landscapes found in the dataset.

**Table 2:** Fidelity scores of the species present in the FSE dataset. Species were ranked according to their fidelity to the arable environment. Here the top and bottom ten species by their ranking are shown. The ecological strategy of these species according to Grime et al. (2014) is also shown, where C species are competitors, S species are stress tolerators and R species are ruderals

Species name	Fidelity score	Fidelity ranking	Grimes’ ecological strategy
*Viola arvensis*	0.560	1	R
*Chenopodium album*	0.555	2	CR
*Sonchus *sp.	0.513	3	R/CR^a^
*Matricaria *sp.	0.501	4	R^b^
*Fallopia convolvulus*	0.498	5	R/CR
*Capsella bursa‐pastoris*	0.492	6	R
*Veronica persica*	0.489	7	R
*Lamium purpureum*	0.452	8	R
*Urtica urens*	0.434	9	R/CR
*Persicaria maculosa*	0.402	10	R/CR
*Cerastium fontanum*	−0.043	172	R/CSR
*Plantago lanceolata*	−0.044	173	CSR
*Fraxinus excelsior*	−0.045	174	C/SC
*Anthriscus sylvestris*	−0.046	175	C/CR
*Urtica dioica*	−0.058	176	C
*Festuca rubra*	−0.066	177	CSR
*Agrostis stolonifera*	−0.071	178	CR
*Dactylis glomerata*	−0.072	179	C/CSR
*Rubus fruticosus *	−0.088	180	SC
*Holcus lanatus*	−0.088	181	CSR

^a^Strategy for *Sonchus oleraceus*.

^b^Strategy for *Matricaria perforata* (Merat).

### 3.3 Species competitiveness

**Figure 2:**
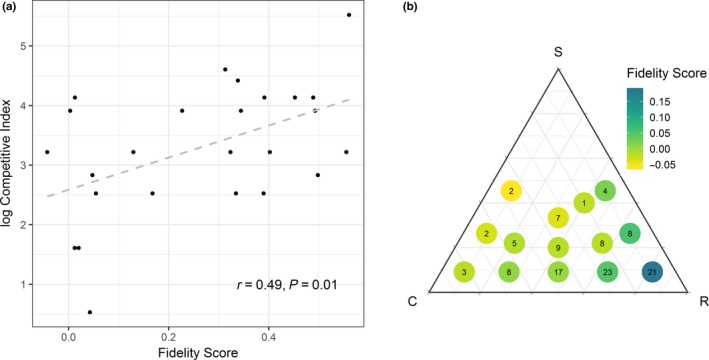
Relationships between species competitiveness and fidelity scores. (a) Correlation between fidelity score and competitive index (number of individuals required to give 5% crop yield loss in wheat; Marshall et al., 2003). (b) Median fidelity score for species showing different ecological strategies according to Grime et al. (2014). The relative position of the circle indicates the ecological strategy, the colour of the circle represents the median fidelity score for that ecological strategy and the number within the circle is the number of species represented by that ecological strategy

We observed a significant correlation between the log competitive index and the fidelity score for the 25 species present in both the FSE and Marshall et al. (2003) datasets (*r* = 0.49, *p* = 0.01, Pearson correlation; Figure 2a). The correlation was positive indicating that species with low fidelity scores require fewer individuals to cause 5% yield loss and so are more competitive.

The 118 species present in our dataset for which ecological strategies were listed by Grime et al. (2014) comprised 14 different ecological strategies. Most of the species were R, R/CR or CR strategists with no S‐strategy species present within the dataset and very few S/R species (the functional group adapted to lower soil fertility that has declined because of the increased use of inorganic fertilisers; Storkey, Moss, & Cussans, 2010) (Figure 2b). There were significant differences between fidelity scores for the different ecological strategies (*F*_(13,102)_ = 4.58, *p* < 0.001) with R strategists having the highest fidelity score. Fidelity scores generally decreased as strategies tended towards C and S types (Figure 2b, Table 2).

### 3.4 Generalised linear mixed effects models

#### 3.4.1 Diversity and abundance

**Figure 3:**
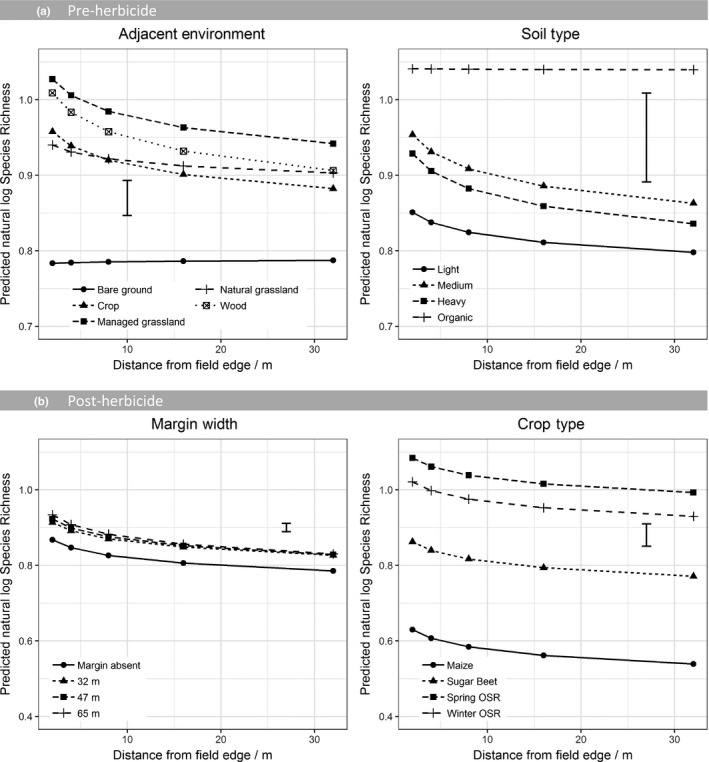
Predicted natural log species richness from a GLMM on (a) the pre‐herbicide dataset and (b) post‐herbicide dataset. Model terms are shown in Table 3. Predictions are classified by distance from field edge and the main effects included in the final model. Predictions are averaged over all other terms included in the model. Error bar shows the approximate average standard error of difference

In all three datasets, there was a consistent effect of distance into the field on species richness (Table 3). In all cases, this represented an increased species richness at the edge of the field with lower species richness at the field centre. For the pre‐herbicide dataset (Figure 3a) and winter wheat dataset (Figure S6) (both assessed prior to the application of contact herbicides), the rate of species richness decline into the field was modified, dependent on the environment in the adjacent parcel of land. Fields adjacent to grassland showed the highest level of overall species richness. The rate of decline in species richness from the field edge to the centre was steepest for fields adjacent to woodland and managed grasslands while there is no increase in species richness at the field edge for fields adjacent to bare ground. For the pre‐herbicide dataset, the species richness was particularly low when adjacent to bare ground. However, in winter wheat, this distinction was not observed. Soil type also significantly impacted species richness showing that the in‐field environment is still very important, although fields with organic soils (which exhibit a different response to other soil types) were underrepresented in the dataset. Crop type was not a significant term explaining variance in weed diversity prior to the application of contact herbicides.

**Table 3:** Final GLMM model terms and their significance (NS term included in model but not significant, *p ≤0.05, **p ≤ 0.01, ***p ≤ 0.001) for all diversity metrics and CWM fidelity scores. A separate model was fitted to each dataset for each response variate. For models fitted to the counts made in winter wheat, the crop type indicated here is the crop in the experimental year and so represents the crop grown in the year prior to the count

Diversity metric	Dataset	Main effects								Interaction with distance into the field						
		Distance into crop	Adjacent environment	Adjacent boundary	Margin presence	Margin Width	Soil type	Field size	Crop type	Adjacent Environment	Adjacent boundary	Margin presence	Margin Width	Soil type	Field size	Crop type
Species richness	Pre‐herbicide	***	NS				NS			***				***		
	Post‐herbicide	***			NS	NS			***			NS	*			
	Winter wheat	***	***	NS			**			**	***			**		
Abundance	Pre‐herbicide	***	***	***	NS		NS		***		**	**		NS		***
	Post‐herbicide	***	NS	NS	NS	NS	NS		*	**	***	*	***	*		***
	Winter wheat	***	***	NS	NS	**				***	**					
Fidelity score	Pre‐herbicide	***	*	**	NS	NS			*	**	***	*	**			
	Post‐herbicide	***			NS		*					**				
	Winter wheat	***		NS	NS		NS		NS		***	**		**		*

However, following the application of herbicide, the role of the adjacent environment became insignificant and instead a significant effect of the crop type was observed indicating the importance of herbicide selectivity; OSR crops supported the largest number of species, whereas maize crops had the lowest species richness (Figure 3b). There was also a small interaction between the margin's width, if present, and the distance into the field. In this case, fields with very wide margins had higher species richness at the field edge with species richness declining towards the centre to reach the same level as in fields with a narrow margin.

**Figure 4:**
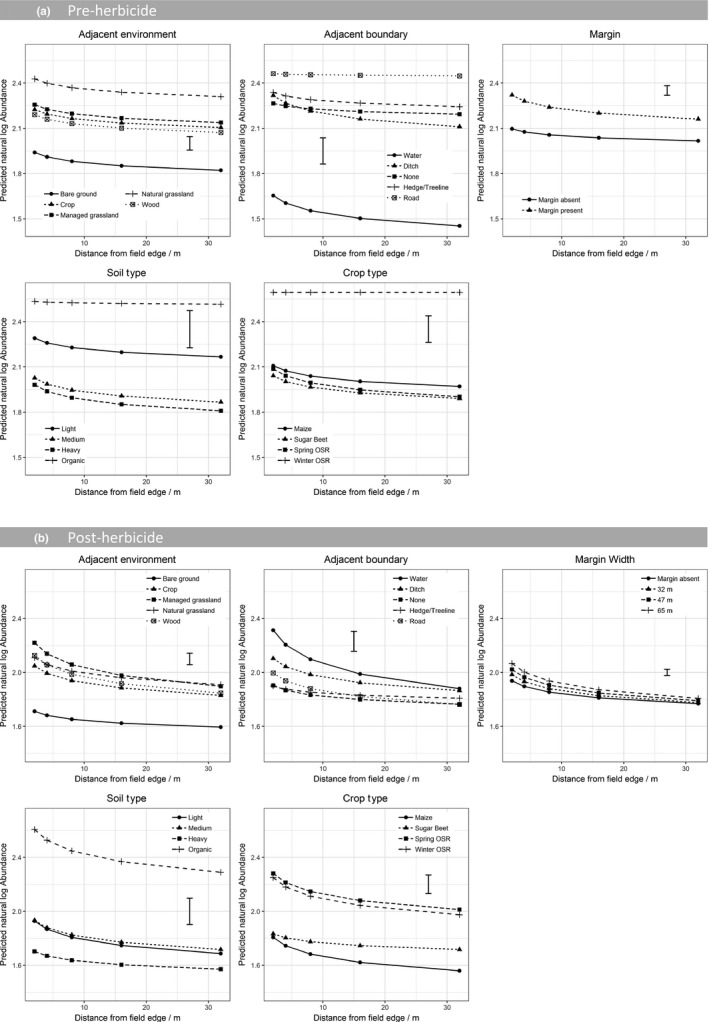
Predicted natural log abundance from a GLMM on (a) the pre‐herbicide dataset and (b) post‐herbicide dataset. Model terms are shown in Table 3. Predictions are classified by natural logarithms of distance into field and all main effects included in the final model. Predictions are averaged over all levels of other terms included in the model. Error bar shows the approximate average standard error of difference

Across all three datasets many terms were selected as being important in determining overall weed abundance (Table 3) indicating the importance of both management (crop type) and environment (soil type) as well as other landscape factors (adjacent environment, adjacent boundary, margin presence and width). In both the pre‐ and post‐herbicide counts, weed abundance differed significantly according to crop type with more individuals counted in OSR crops (Figure 4). Both before and after herbicide application, fields adjacent to bare ground had the lowest weed abundance and fields next to grassland the highest (Figure 4). In winter wheat, abundance was particularly high at the field edge in fields adjacent to grassland (Figure S7). Weed abundance also significantly varied with different boundary types although the response was not consistent across all three datasets (Figure 4, Figure S7). Fields adjacent to roads or farm tracks had the greatest weed abundance prior to herbicide application with high abundance of weeds at all distances into the field.

#### 3.4.2 CWM fidelity scores

**Figure 5:**
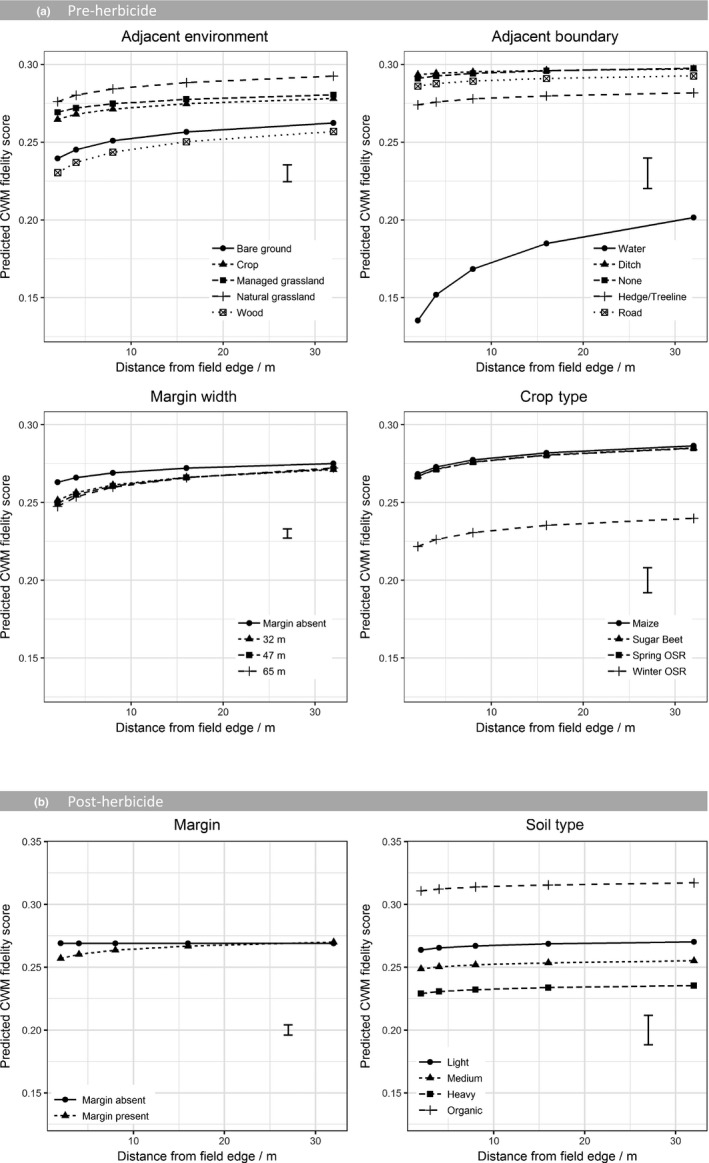
Predicted CWM fidelity score from a GLMM on (a) the pre‐herbicide dataset and (b) post‐herbicide dataset. Model terms are shown in Table 3. Predictions are classified by natural logarithms of distance into field and all main effects included in the final model. Predictions are averaged over all levels of other terms included in the model. Error bar shows the approximate average *SE* of difference

The CWM fidelity scores increased towards the field centre (Figure 5). This indicates that species present in the centre of the field are those more typical of arable habitats (resident species) while those present at the edge of the field are more likely to originate from other habitats (transient species). As well as having higher abundance, fields neighbouring grassland also had plant communities with the highest fidelity to arable habitats indicating that species originating from grassland were most likely to be able to colonise arable fields. Prior to herbicide application (pre‐herbicide and winter wheat datasets), the adjacent boundary feature showed a significant interaction with distance into the field indicating that the way in which CWM fidelity scores changed from the field edge to the centre was modified by the boundary feature (Figure 5, Figure S8). Fields adjacent to a watercourse had communities with the lowest fidelity scores and the steepest gradient in CWM fidelity score from the field edge to the centre, whereas fields with no boundary features tended to show little change in fidelity between the field edge and centre.

Following the application of herbicide, the difference in fidelity score at the field edge was largely driven by the presence of a margin and the distance into the field. At the field centres, fidelity scores were generally high with communities composed of ‘arable species’. When there was no margin this stayed the same from the field centre to the edge, yet when there was a margin present, the fidelity scores dropped at the field edge indicating that a proportion of the individuals colonising the cultivated field from the margin were able to persist following the application of contact herbicide.

## 4 DISCUSSION

Our results confirm the importance of the immediate landscape in influencing the increased weed diversity and abundance observed at field edges. This provides evidence for the hypothesis that spatial mass effects contribute significantly to in‐field plant diversity and abundance and that weed communities are composed of both resident weed communities, replenished by the in‐field seed bank and transient communities, which rely on repeated colonisation of field edges. We demonstrated that the spatial distribution of species richness at the field scale, namely a decline in diversity with distance into the field, is highly dependent on the immediate landscape context. This confirms our first hypothesis that there is a significant interaction between the decline in diversity with distance into the field and the nature of the neighbouring habitat and/or boundary feature.

The highest species richness and abundance prior to the application of contact herbicides was observed in fields adjacent to grasslands. This is indicative of grassland species having high potential to colonise arable fields and is also reflected in the relatively high CWM fidelity scores for fields neighbouring grassland. This was in contrast to the low species richness observed in fields adjacent to bare ground (including urban) where there is a limited source of new species in the local environment. Notably for the 135 transects next to bare ground, no decline with distance into the field was observed, implying that spatial mass effects may be exclusively responsible for the increased species richness at the edges of intensively managed fields.

It is interesting to note the differences between managed and natural grassland systems in terms of their effect on the total weed abundance as well as the gradient of species richness in the adjacent crop. In the pre‐herbicide dataset, we observed more species overall and a steeper gradient in species richness from managed grasslands to the field centres, whereas the gradient from natural grassland to the field centres was shallower (although this difference was not seen in the winter wheat dataset). Natural grasslands, which generally have lower soil fertility, largely consist of relatively less competitive stress‐tolerant species (characterised by a slow growth rate and low specific leaf area) that are likely to be less well adapted to the highly fertilised cropped field edge (DeVries et al., 2012). The prevalence of vegetative regeneration traits in natural grasslands and greater amounts of seed dispersal in managed grasslands (Pakeman, 2004) also helps to explain the higher spatial mass effects from managed grasslands.

Our second hypothesis that we would see an increase in fidelity to arable field habitats from the field edge to the centre was also confirmed by our analyses indicating that transient communities at the field edge are less typical of the arable environment, whereas it is the resident communities, comprising typical arable species, that are found at the field centre. The steepest declines in diversity were observed next to woodland, which would act as a source of species poorly adapted to disturbed arable fields—a conclusion also supported by the low fidelity scores observed in these transects. The idea that field boundaries are acting both as an additional source for spatial mass effects and as a barrier to dispersal is also supported by our analysis of fidelity scores, as fields adjacent to water have the lowest abundance and communities with the lowest fidelity scores possibly due to the large difference in species composition of wetlands compared to the arable field. The observation that the presence of field margins can lead to increased abundance in the field and reduced fidelity scores indicates that they may have a similar effect as neighbouring grassland (reflecting the fact that field margins in the UK are dominated by grass buffer strips). This supports the results of Marshall (2009) who found that grass margins can be a source of grasses, such as *Festuca rubra*, colonising the cropped field.

The reduction in species richness post‐herbicide, and the associated reduction in the number of landscape factors explaining that species richness, supports our third hypothesis and demonstrates how the application of herbicide is effective in removing transient species (rare weeds and species ingressing from other habitats; Gaba, Gabriel, Chadœuf, Bonneu, & Bretagnolle, 2016). However, resident weed species that are present in high numbers in the centre of fields can persist post‐herbicide application owing to buffering from large persistent seedbanks and also, possibly, evolved resistance to herbicides (Neve, Vila‐Aiub, & Roux, 2009). The importance of crop type in determining species richness post‐herbicide is likely to be an artefact of herbicide efficacy and selectivity in those crops and again supports the idea that the communities present at this stage are dominated by the resident weed communities and many transient species have been effectively removed by the herbicide.

Our analysis gives strong support for the view that increased species richness at the edges of fields is largely a result of spill‐over from neighbouring habitats and that spatial mass effects are a key process explaining increased weed diversity and abundance at the edges of conventionally managed fields. The absence of any S or S/R species from our dataset or any rare weeds (on the UK Biodiversity Action Plan, 2018 list of rare species in the UK) highlights the fact that intensive agriculture has dramatically depleted the arable flora in much of the arable landscape and so conservation measures should be targeted at areas where high diversity still remains (Albrecht et al., 2016). We also found that decreasing fidelity scores (associated with the field edge) were linked to more competitive species, meaning the transient weed community is more competitive in nature, and ecologically distinct from the ruderal (R) dominated resident community. This finding has important implications for how we view field edges in terms of their potential to conserve arable plant communities in conventionally managed fields. While there was evidence that the competitive transient species were being effectively controlled with herbicides, if left unchecked (in the absence of herbicides) they could become problematic weeds—lower fidelity scores were correlated with a lower competitive index (fewer individuals required to give 5% yield loss).

The common weed flora has an important role in supporting farmland biodiversity (Bretagnolle & Gaba, 2015; Marshall et al., 2003) and ruderal species have been shown to disproportionately provide resources for phytophagous insects as well as providing chick food (Storkey et al., 2013). The seeds of many ruderal species are also an important component in the diet of farmland birds (Eraud et al., 2015; Gaba, Collas, Powolny, Bretagnolle, & Bretagnolle, 2014). Perennial field margins provide a habitat to support farmland biodiversity which may offset the habitats being lost through the conversion of semi‐natural grasslands. However, these margins do not provide an opportunity for ruderal species, which require areas of natural regeneration, to persist (Butler et al., 2009). Recommendations for conserving arable plant diversity and supporting the ecosystem services provided by the ruderal flora include reducing fertiliser and herbicide application at the field edge (Albrecht et al., 2016; Wagner et al., 2017). A land‐sparing approach where these ‘conservation headlands’ are maintained on conventionally managed farms would help restore plant diversity to similar levels to those found in organic farms (Fuller et al., 2005). Our results highlight the importance of considering the neighbouring habitat and boundary when deciding where to place these options in the landscape. Where there is a danger of competitive species colonising a conservation headland (i.e. adjacent to managed grasslands or margins), they could become dominant in the absence of herbicides. These more competitive species would suppress the desirable ruderal species and potentially become problematic for crop production within the field. As such, the success of these conservation measures will depend on the immediate landscape context, and the potential ingress of competitive species should be considered when deciding on their arrangement in the farm landscape and subsequent management.

## AUTHORS’ CONTRIBUTIONS

H.M., S.B. and J.S. conceived the ideas. H.M. and K.H. designed the analysis and analysed the data. H.M. led the writing of the manuscript. All authors contributed critically to the drafts and gave final approval for publication.

## Data Availability

FSE data for Spring OSR, winter OSR, beet and maize are available via the Environmental Information Data Centre. https://doi.org/10.5285/0023bd6e-4dd7-462c-aacf-f13083b054ab (Scott et al., 2012). https://doi.org/10.5285/37a503da-d75c-4d72-8e8b-b11c2fdc7d92 (Scott et al., 2012). https://doi.org/10.5285/86cd1a60-64f1-4087-a9f1-a3d8a9f8f535 (Scott et al., 2012). https://doi.org/10.5285/ca6752ed-3a22-4790-a86d-afadaedda082 (Scott et al., 2012). Countryside Survey data are available via the Environmental Information Data Centre https://doi.org/10.5285/57f97915-8ff1-473b-8c77-2564cbd747bc (Bunce et al., 2014).
