# Supplementary material for: The contribution of spatial mass effects to plant diversity in arable fields
Source: J Appl Ecol. 2019 Jun 3;56(7):1560–74. doi: 10.1111/1365-2664.13414 (PMC6618144; doi:10.1111/1365-2664.13414)
Supplement: Supplementary file 1 [file JPE-56-1560-s001.docx]

# Supplementary Information

| a)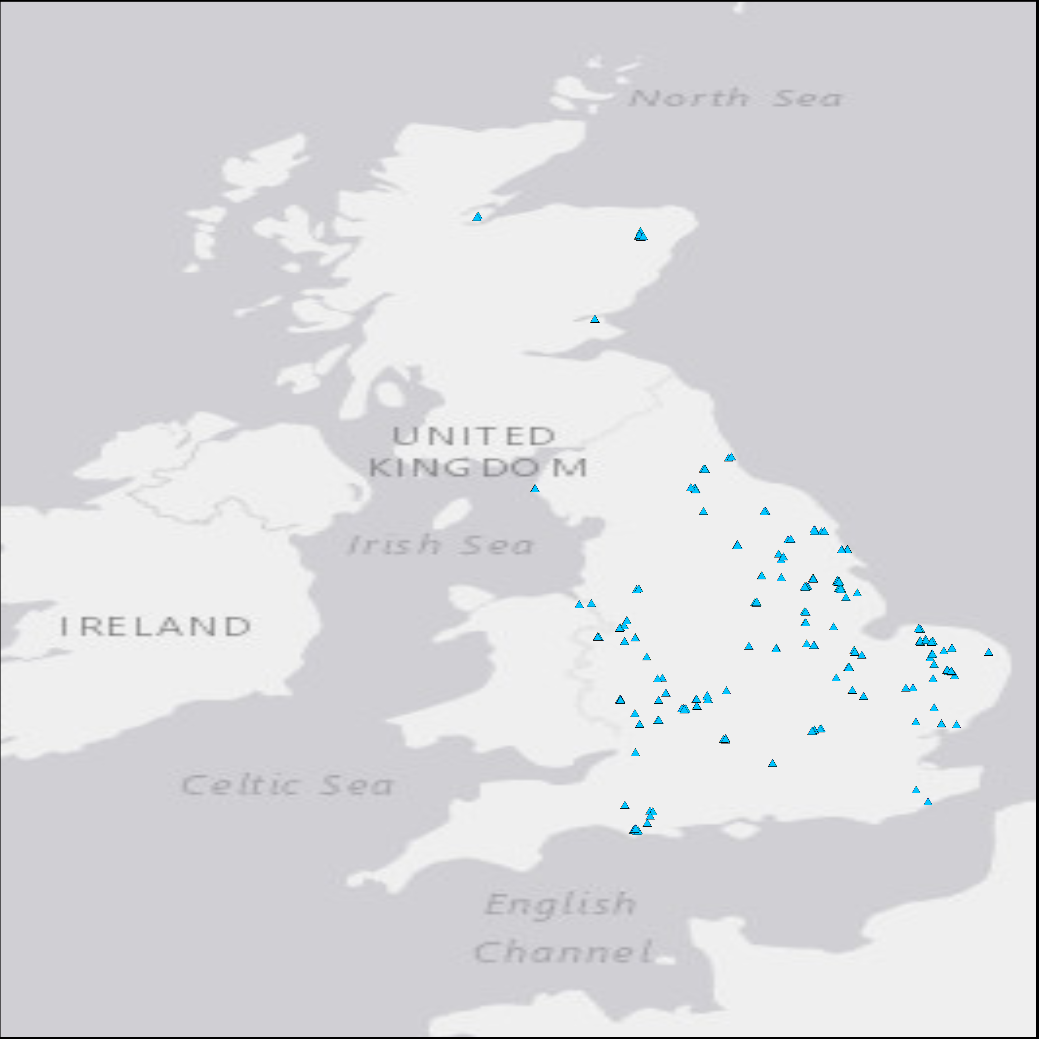 | b)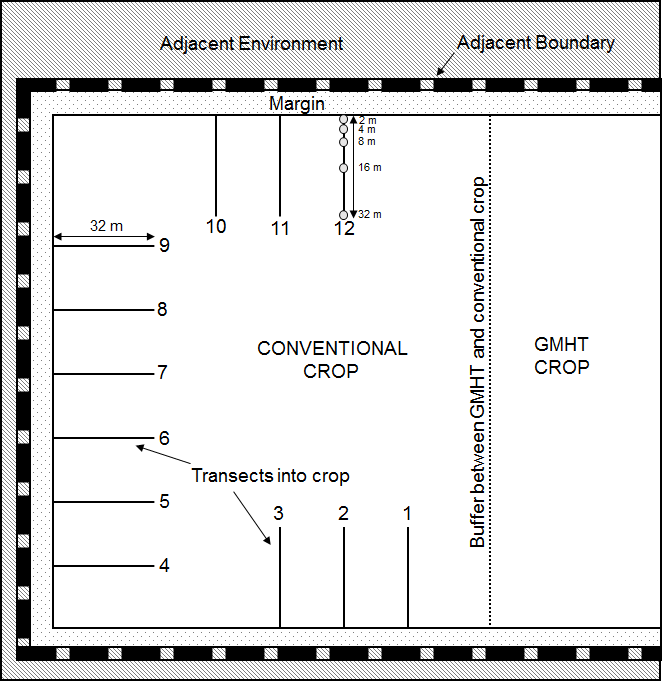 |
| --- | --- |

Figure S1: Sites included in the Farm Scale evaluations (FSE) study of genetically modified herbicide tolerant (GMHT) crops. a) Location of the farms included in the FSE. The location of each farm is represented by a blue triangle. b) The experimental layout of the field sites included in the FSE dataset. Each field was divided into a GMHT and a conventional half field (here we excluded all data from the GMHT halves). 12 transects of 32m were placed in the positions indicated by the numbers 1-12, with sampling points at 2,4,8, 16 and 32m on each transect. The presence and width of a margin, and the type of adjacent boundary and adjacent environment was recorded at the end of each transect. (Figure S1b adapted from Heard et al., 2003)

**
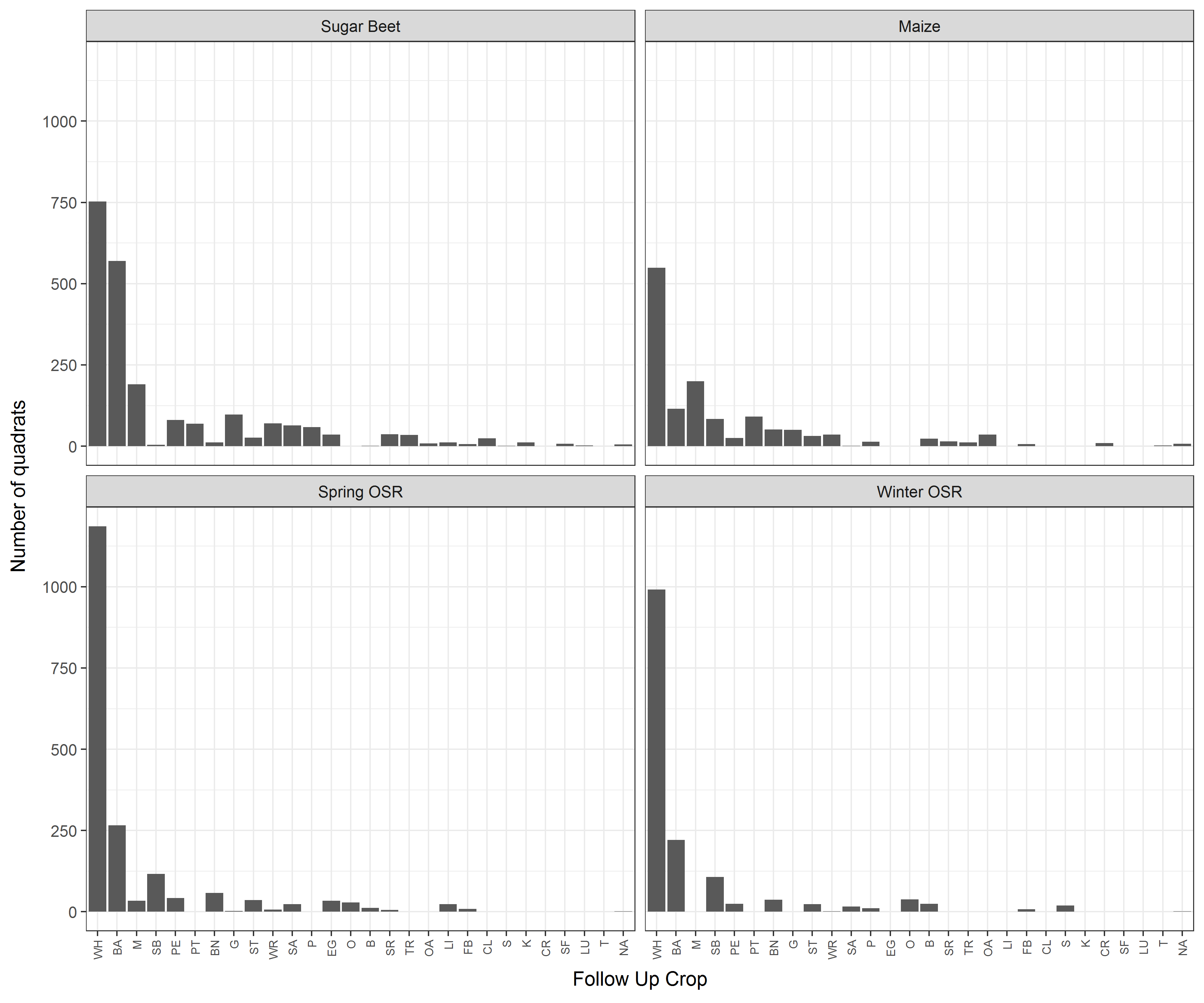
**

Figure S2: Crops grown at more than one site in the year following the experimental crop of (a) sugar beet, (b) maize, (c) spring OSR, and (d) winter OSR. Data are presented at the quadrat level as some sites were split in the follow-up year and had different crops grown on different parts of the site. The experimental crops are separated into separate panels. WH=wheat, BA=barley, M=maize, SB=sugar beet, PE=pea, PT=potato, BN=beans, G=grass, ST=stubble, WR=winter OSR, SA=set aside , P=ploughed no crop, EG=elephant grass, O=barley + Lolium, B=beet, SR=spring OSR, TR=triticale, OA=oats, LI=linseed, FB=fodder beet, CL=clover, S=sprayed dead, K=kale, CR=cress, SF=sunflower, LU=lupins, T=trees, NA=no information on follow-up crop


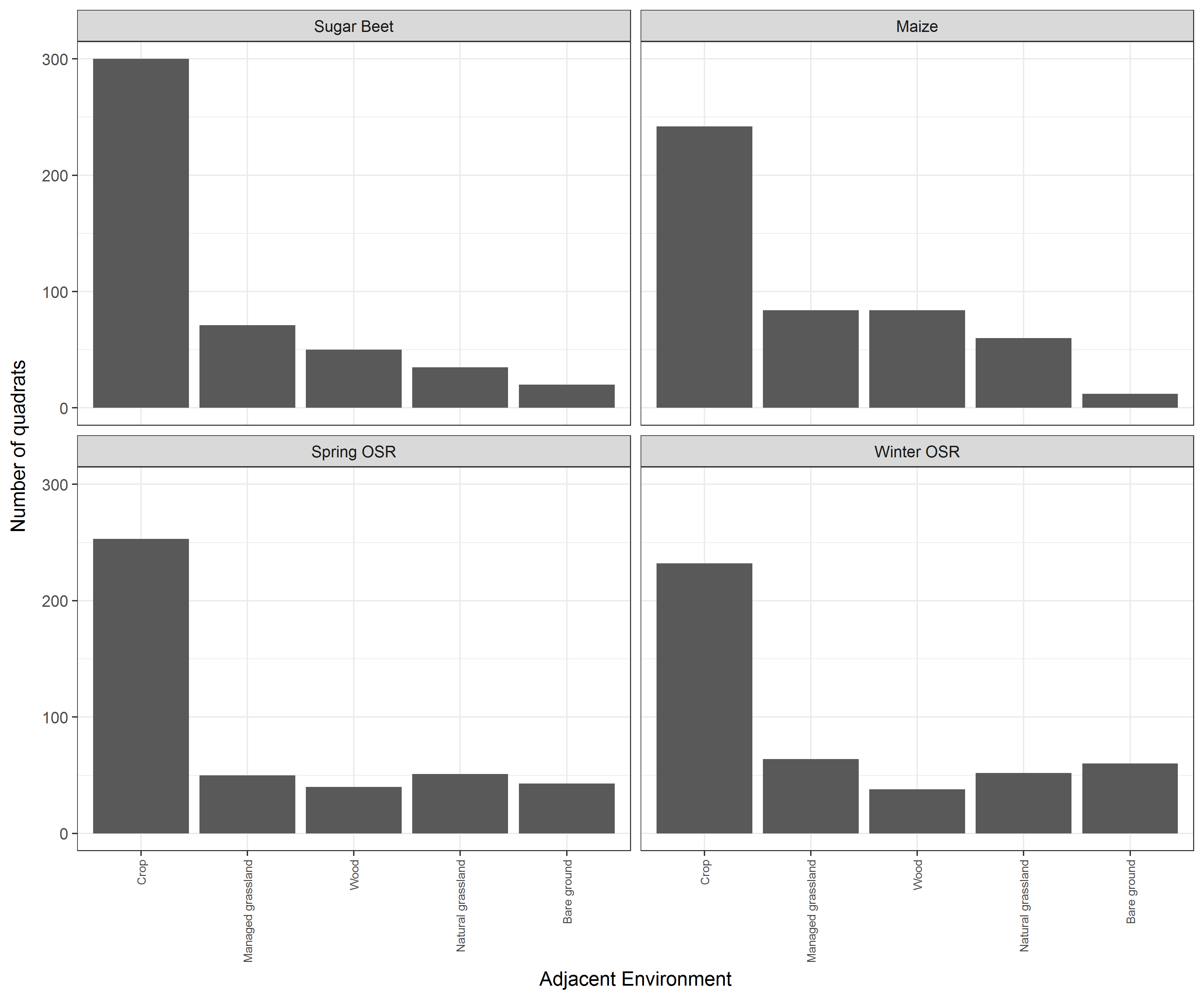


Figure S3: The number of occurrences of each type of adjacent environment. Panels are grouped according to the crop grown (a) sugar beet, (b) maize, (c) spring OSR, and (d) winter OSR. Data are presented at the quadrat level as some sites had different environment types adjacent to different parts of the field edge. Adjacent environments are grouped into Crop, Managed grassland (fertile agricultural grassland or newly sown grassland), Wood (woodland or belt of trees), Natural grassland, and Bare ground (urban or ploughed field). In total, 1027 quadrats were on transects where the adjacent environment was crop, 269 were adjacent to managed grassland, 212 were adjacent to wood, 198 were adjacent to natural grassland and 135 were adjacent to bare ground.


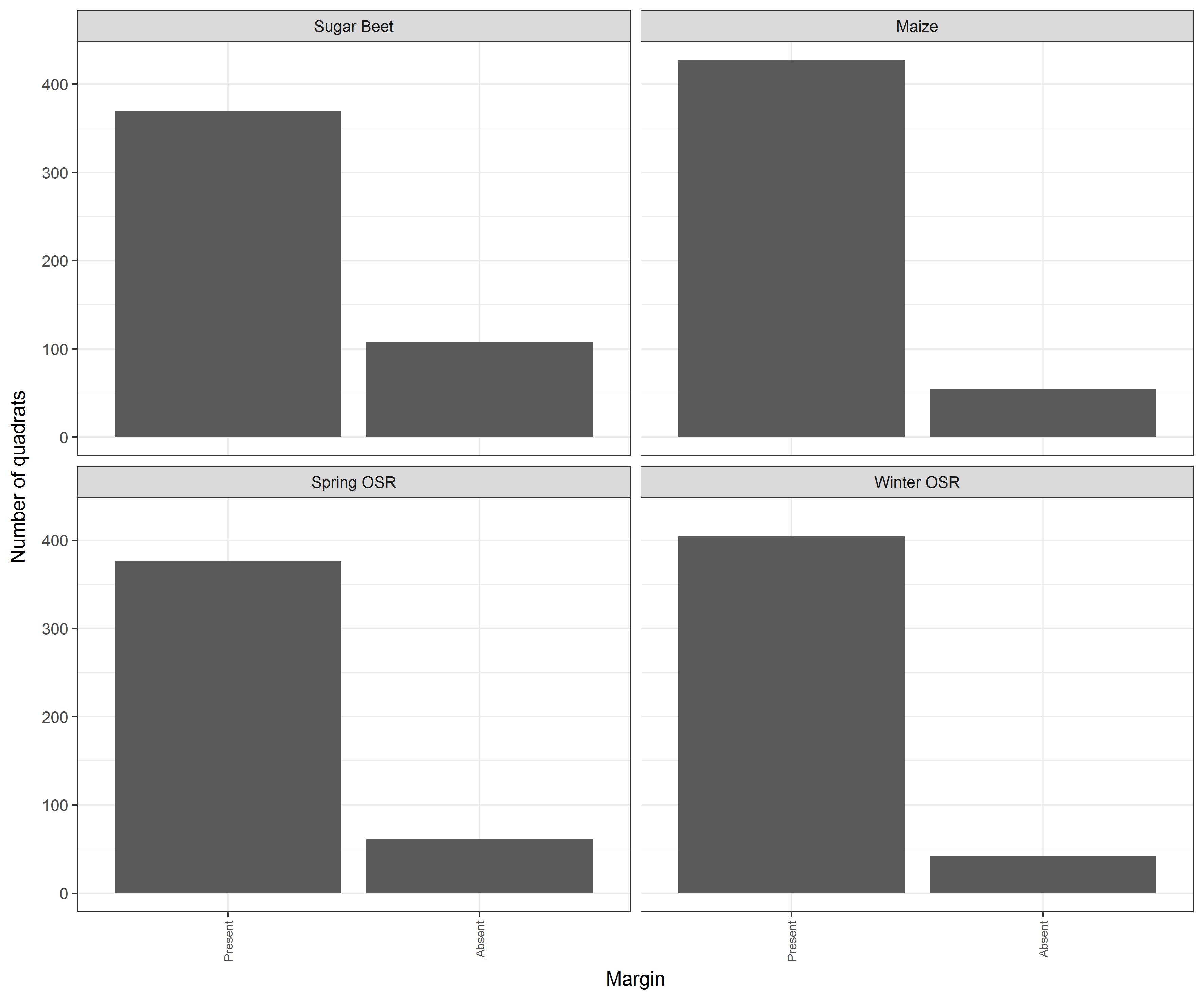


Figure S4: The number of occurrences of a margin. Panels are grouped according to the crop grown (a) sugar beet, (b) maize, (c) spring OSR, and (d) winter OSR. Data are presented at the quadrat level as some sites had margins adjacent to only some parts of the field edge.


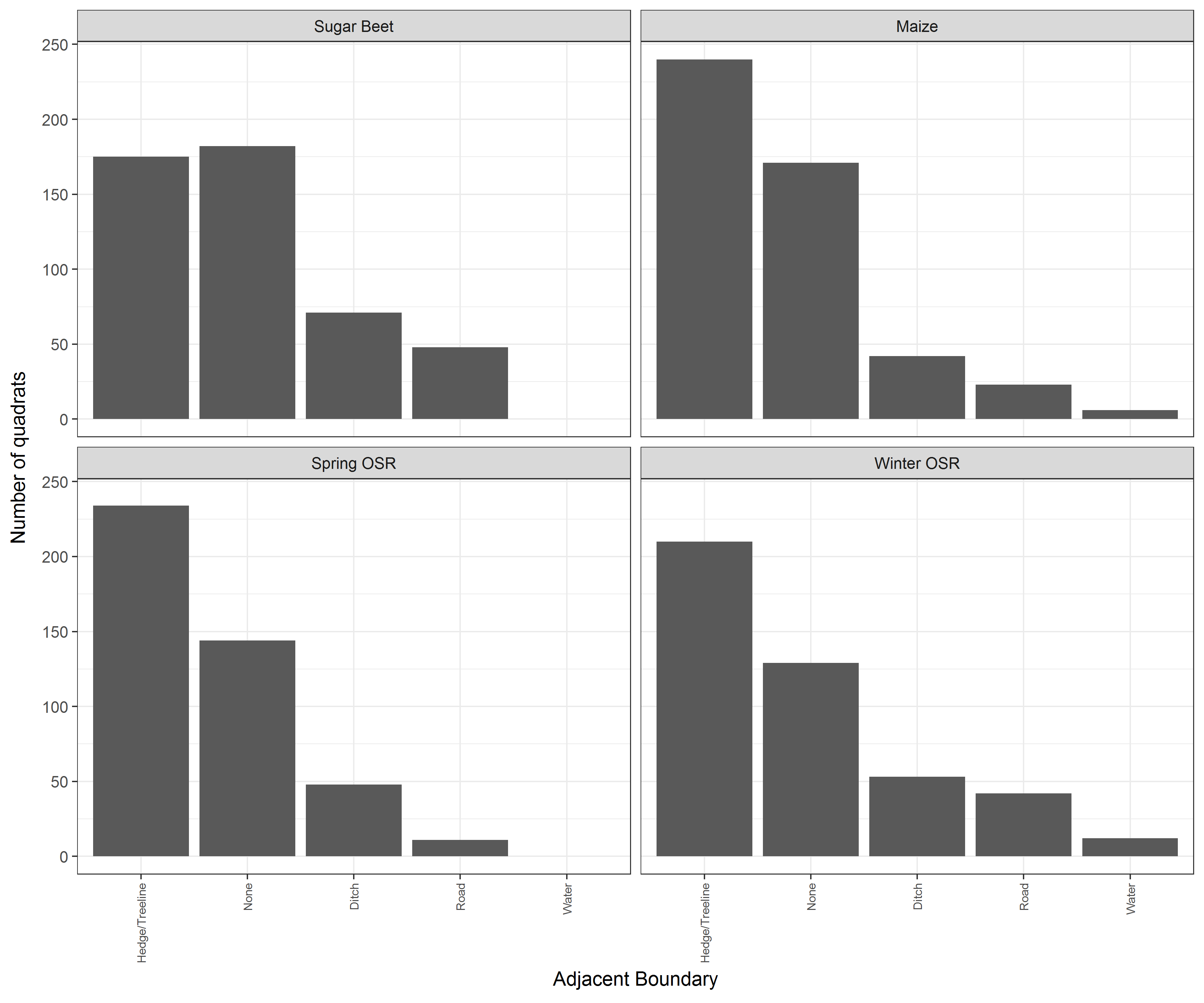


Figure S5: The number of occurences of each type of adjacent boundary. Panels are grouped according to the crop grown (a) sugar beet, (b) maize, (c) spring OSR, and (d) winter OSR. Data are presented at the quadrat level as some sites had different boundaries adjacent to different parts of the field edge. Adjacent boundaries are grouped into Hedge/treeline, None, Ditch, Road (road or vehicle track) and Water (pond or stream). In total, 859 quadrats were on transects adjacent to a hedge/treeline, 626 had no adjacent boundary, 214 were adjacent to a ditch, 124 were adjacent to a road and 18 were adjacent to water.

Table S1: Number of quadrats with each combination of factor levels for all 2-way factor combinations. We did not include these two-way interactions in our analyses due to the high levels of imbalance and the presence of combinations for which there were no data (11 out of157).

|  |  | Adjacent Boundary | | | | | Margin | | Soil Type | | | | Crop | | | |
| --- | --- | --- | --- | --- | --- | --- | --- | --- | --- | --- | --- | --- | --- | --- | --- | --- |
|  |  | None | Road | Water | Hedge or treeline | Ditch | Absent | Present | Heavy | Light | Medium | Organic | Sugar Beet | Maize | Spring OSR | Winter OSR |
| Adjacent Environment | Crop | 1204 | 317 | 32 | 1892 | 603 | 609 | 3439 | 973 | 356 | 1873 | 270 | 1191 | 908 | 919 | 1030 |
|  | Natural Grassland | 351 | 44 | 0 | 384 | 36 | 119 | 696 | 173 | 103 | 410 | 53 | 150 | 238 | 178 | 249 |
|  | Managed Grassland | 536 | 69 | 0 | 509 | 31 | 189 | 956 | 448 | 113 | 398 | 28 | 307 | 344 | 189 | 305 |
|  | Bare | 200 | 3 | 23 | 268 | 53 | 131 | 416 | 154 | 26 | 284 | 15 | 67 | 42 | 176 | 262 |
|  | Wood | 328 | 65 | 25 | 415 | 19 | 8 | 844 | 119 | 19 | 521 | 30 | 171 | 366 | 148 | 167 |
| Adjacent Boundary | None |  |  |  |  |  | 833 | 1786 | 470 | 298 | 1351 | 119 | 737 | 679 | 620 | 583 |
|  | Road |  |  |  |  |  | 0 | 498 | 85 | 23 | 278 | 0 | 183 | 90 | 21 | 204 |
|  | Water |  |  |  |  |  | 0 | 80 | 38 | 0 | 25 | 0 | 0 | 28 | 0 | 2 |
|  | Hedge or treeline |  |  |  |  |  | 213 | 3255 | 998 | 284 | 1690 | 128 | 744 | 922 | 844 | 958 |
|  | Ditch |  |  |  |  |  | 10 | 732 | 276 | 12 | 142 | 149 | 222 | 179 | 125 | 216 |
| Margin | Absent |  |  |  |  |  |  |  | 12 | 132 | 634 | 53 | 398 | 192 | 285 | 181 |
|  | Present |  |  |  |  |  |  |  | 1685 | 485 | 2852 | 343 | 1488 | 1706 | 1325 | 1832 |
| Soil Type | Heavy |  |  |  |  |  |  |  |  |  |  |  | 249 | 72 | 312 | 514 |
|  | Light |  |  |  |  |  |  |  |  |  |  |  | 452 | 0 | 96 | 69 |
|  | Medium |  |  |  |  |  |  |  |  |  |  |  | 1063 | 900 | 93 | 600 |
|  | Organic |  |  |  |  |  |  |  |  |  |  |  | 122 | 91 | 183 | 0 |

| 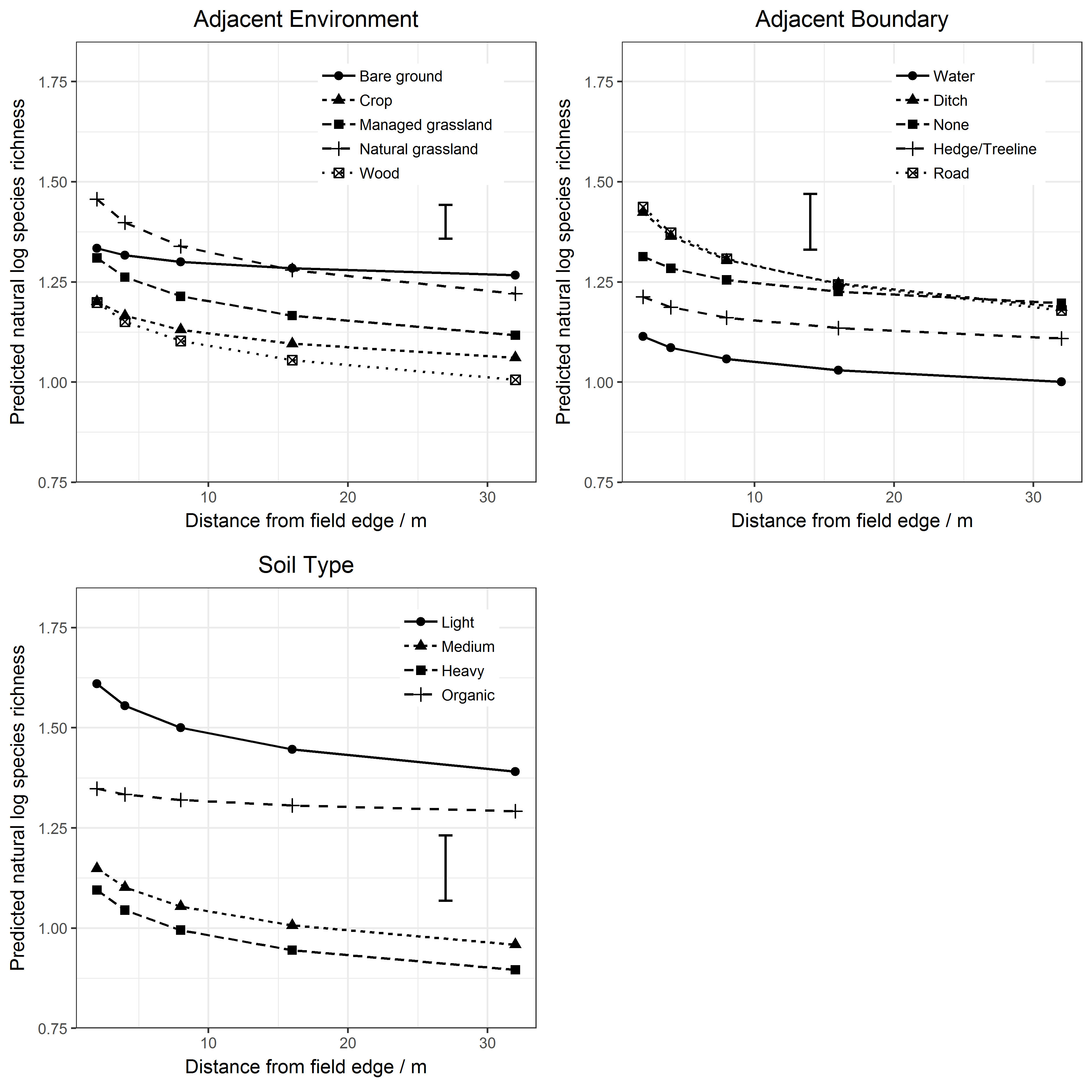 |
| --- |
| Figure S6: Predicted natural log species richness from a GLMM on the follow up dataset. Model terms are shown in Table 3. Predictions are classified by natural logarithms of distance into field and all main effects included in the final model. Predictions are averaged over all levels of other terms included in the model. Error bar shows the approximate average standard error of difference (calculated on variance scale). |
|  |
| 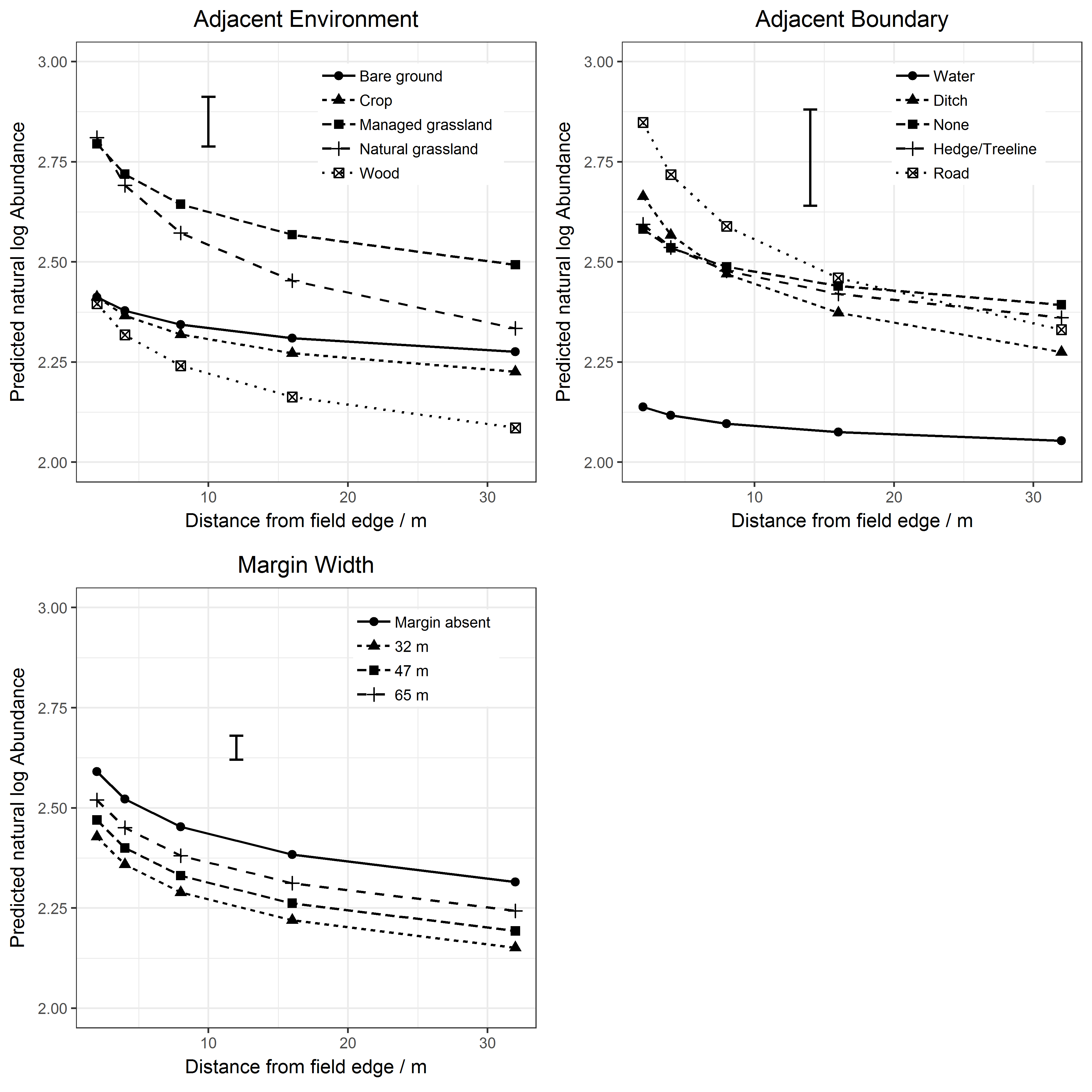 |
| Figure S7: Predicted natural log abundance from a GLMM on the follow up dataset. Model terms are shown in Table 3. Predictions are classified by natural logarithms of distance into field and all main effects included in the final model. Predictions are averaged over all levels of other terms included in the model. Error bar shows the approximate average standard error of difference (calculated on variance scale). |

| 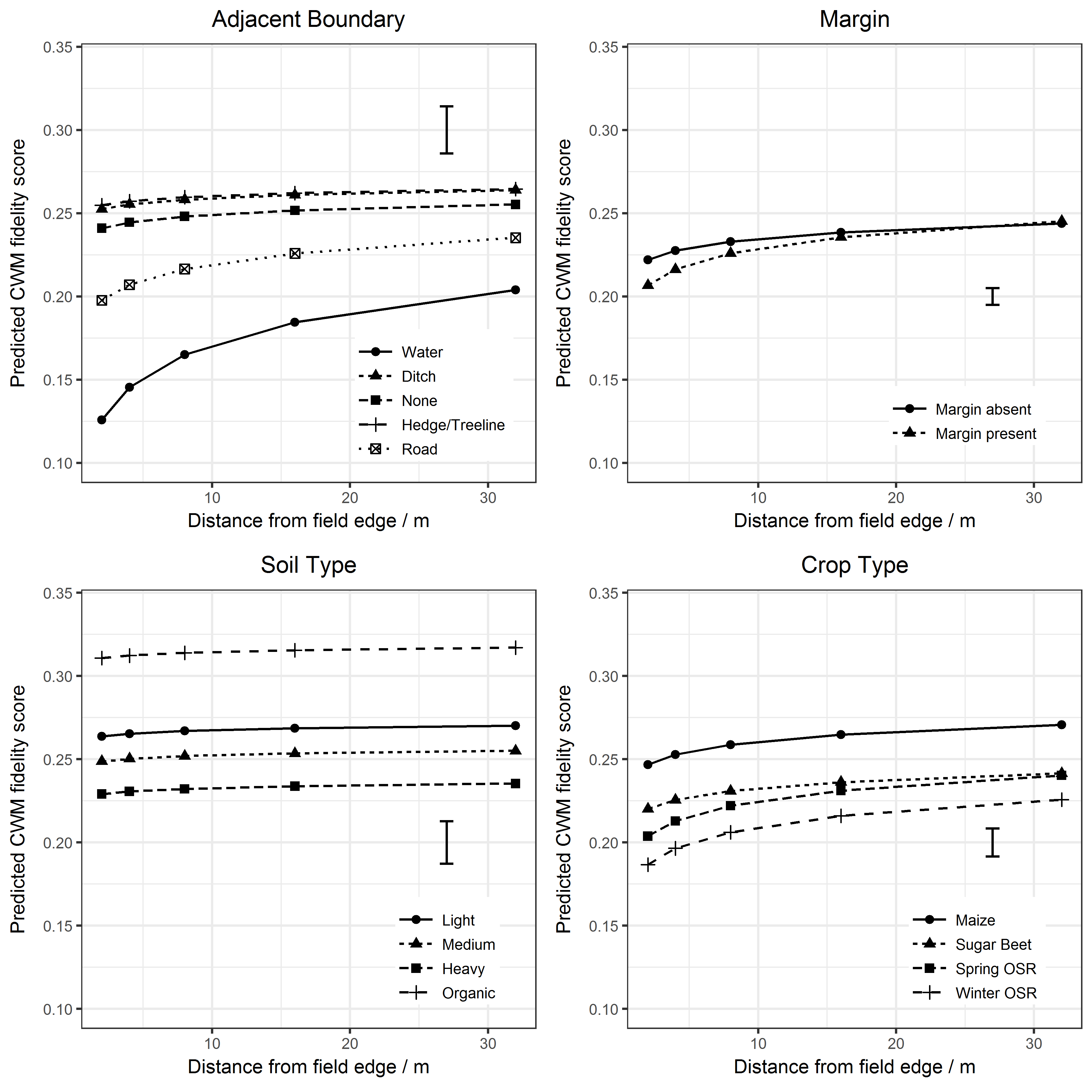 |
| --- |
| Figure S8: Predicted CWM fidelity score from a GLMM on the follow up dataset. Model terms are shown in Table 3. Predictions are classified by natural logarithms of distance into field and all main effects included in the final model. Predictions are averaged over all levels of other terms included in the model. Error bar shows the approximate average standard error of difference (calculated on variance scale). |
